# Inter- and Intra-racial Differences in Pain and Performance-Based Function in Older Adults With Osteoarthritis

**DOI:** 10.1093/geroni/igab046.3216

**Published:** 2021-12-17

**Authors:** Staja Booker, Roger Fillingim

**Affiliations:** 1 University of Florida, GAINESVILLE, Florida, United States; 2 University of Florida, gainesville, Florida, United States

## Abstract

Osteoarthritis (OA) contributes to movement-evoked pain, impaired function and mobility, and reduced quality of life among older adults. Assessment of pain has not traditionally considered the dynamic changes that occur with gross motor movement, and thus self-reports of pain often reflect static or resting pain. This case-control pilot study examined inter- and intra-racial differences in movement-evoked pain and performance-based function in older adults (N= 28) with knee OA. Cases consisted of Blacks and Whites with OA; controls included Blacks without OA. The Biodex Pro System 4, an isokinetic and isometric dynamometer commonly used in rehabilitative medicine, measured knee muscle function. Pain intensity was assessed pre-, ante-, and post- completion of 2 repetition sets of five alternating knee flexion and extension maximum voluntary contractions at angular velocities of 90° (greater resistance) and 180° (lower resistance). Repeated Measures Analysis of Variance with Bonferroni correction identified statistically significant differences in pain for within- and between-subjects at 90° and 180°. Pain increased during the repetitions and decreased after completion of both repetition sets; this non-linear relationship was significant (p= .004). One-way ANOVA demonstrated peak torque (extension), a muscle’s maximum strength capability, was significantly higher in White cases and Blacks controls compared to Blacks cases. Novel findings revealed that baseline pain is much higher and functional performance is significantly lower in Blacks with OA compared to White cases and Black controls. This research advances precision pain measurement and our understanding of the biological mechanisms uniquely involved in the experience of knee OA and mobility.

